# Towards interventions on school dropouts for disabled learners amidst and post-COVID-19 pandemic

**DOI:** 10.4102/ajod.v11i0.1009

**Published:** 2022-06-24

**Authors:** Tawanda Makuyana

**Affiliations:** 1 Department of Tourism Research in Economic Environs and Society (TREES), Faculty of Economic and Management Sciences, North-West University, Potchefstroom, South Africa; 2 Department of Research and Development, National Council of and for Persons with Disabilities, Johannesburg, South Africa

**Keywords:** inclusive education, school dropout interventions, COVID-19, underlying obstacles, dropout of disabled pupils

## Abstract

**Background:**

Despite objective arguments for inclusive education, there is a dearth of mechanisms to reduce dropouts amongst disabled learners in the extant literature. Thus, this article is one of the outputs of a study, which was conducted after a consistent observation of dwindling numbers of disabled learners who succeed in basic education in South Africa. Of late, the dropout rate increased because of adherence to lock down regulations amidst the coronavirus disease 2019 (COVID-19) pandemic. This triggered the need for research on co-creating interventions to mitigate the rate of dropouts amongst disabled learners.

**Objective:**

The article explores underlying obstacles that induce school dropouts for disabled learners amidst and post-COVID-19 and postulates interventions accordingly.

**Methods:**

Descriptive-narrative research upheld reality as emerging from empirical experiences of parents and guardians of disabled children, heads of primary and secondary schools, social workers, the Department of Social Development and Basic Education, and provincial associations for disabled persons that focus on children. Lived experience-based opinions were obtained from provinces with different economic growth, namely, Limpopo and Gauteng. Forty-one in-depth one-on-one interviews and two focus group discussions used Google Meet. The collected data were analysed using Creswell’s qualitative data analysis framework (steps) and Atlas.ti.8.

**Results:**

The findings show a consistent pattern that the COVID-19 pandemic exacerbated the parents and guardians’ fear of exposing and risking their learners to the health crisis. Based on the parents and guardians’ narrative, mainstream school administrations discriminate and are unwilling to enrol disabled learners. Furthermore, the narrative from the school leadership shows that teachers use exclusive teaching and learning methods for the enrolled disabled learners because of ignorance, misconception, misunderstanding, misinterpretation of disability, disability inclusion, and reasonable accommodation.

**Conclusion:**

Based on the finding, it is clear that dropouts amongst disabled learners can be alleviated by using a systematic multi-stakeholder local community-based intervention approach. This, therefore, implies that government authorities and agencies should incorporate disability into mainstream policies that guide planning, budgeting, staffing, and mobilisation of other resources. This would ideally enhance the provision of learning opportunities to disabled learners whilst supporting their diverse educational needs without dichotomies set by ‘ability and disability’, or normal and abnormal. In this manner, inclusive education can contribute to the educational success of disabled learners through developing sustainability and resilience amongst disabled learners.

## Introduction

Inclusive education has been an issue of debate for more than 15 years in South Africa. The current article relates to the study conducted by Makuyana (2020) which focused on accessible human capital development because the primary aim is to enhance the educational success of disabled students. One can say that it is a theoretical concept with substantive challenges in application and management, to the extent that the extant literature reflects the complexities beyond being an ‘issue of debate’. Nonetheless, Funda (2020) understood inclusive education as the processes and procedures that continuously address and respond to the needs of diverse learners for improved active involvement in learning and communities’ environment. Within this context, this article considers school dropout (‘withdrawal’ or ‘attrition’) as ‘leaving education without obtaining a minimal credential’ (Funda 2020). The lowest qualification of South African individuals is the National Senior Certificate (NSC) or ‘matric’ (Donohue & Bornman 2014). In most cases, dropouts of non-disabled learners from grades 10 and 11 result in 50% of learners in a cohort reaching grade 12 (Donohue & Bornman,2014). Research in this regard estimated the dropouts to be 750 000 in May 2021 for non-disabled learners only (https://zerodropout.co.za/). Usually, there is a dearth of documentation for disabled learners, whilst non-disabled learners’ records are consistently documented in primary and secondary schools.

The sustainability of inclusive education entails a systemic process concerned with addressing the present needs without compromising the ability of future generations’ educational and societal needs (host communities, vulnerable population groups such as disabled people), planet (environment and biodiversity), and profit-making (Botha 2020; Medina-García, Doña-Toledo, & Higueras-Rodríguez, 2020; Turusida 2020). At the same time, inclusive education can enable learning outcomes that uphold the ability to adjust and recover from a societal, cultural, and social background that could have been prevented or mitigated with sustainable practices whilst perpetuating a decrease in the dropout of disabled learners (Ostendorf 2020). However, there is limited attention to developing proficiencies that provide capacity and capabilities to all teachers in all subjects because of the intersectionality of disability and the need to include diversified learners in times of crisis (COVID-19 included) (Funda, 2020).

Nonetheless, the Zero Dropout Campaign is an initiative of a non-profit organisation (civil society organisation) funded by DG Murray Trust since 2015 to develop a toolkit to prevent dropouts, which was first implemented in 2017 among South African individuals. The said campaign is towards giving attention to non-disabled learners only as it is silent on disabled cohorts, particularly in health-threatening situations (https://zerodropout.co.za/). In addition, there is a dearth of research on dropouts of disabled learners that has disaggregated the statistics by, for example, age, type of impairments, and gender before and amidst the COVID-19 pandemic. On the one hand, the 2014-General Household Survey Report stated that a total of 480 036 disabled learners out of 718 409 were out of school (Statistics South Africa 2014). This means approximately 66% were dropouts. On the other hand, the Department of Basic Education (2016) estimated the number of disabled learners who were out of school to be 597 753 in 2015, thereby showing an increase of 117 717 dropouts amongst disabled learners between 2014 and 2015. This means that 83% of the South African disabled children are out of school. This implies that disabled learners scarcely succeed at par with their non-disabled cohort; for example, the Department of Basic Education-NSC 2018 Examination Report showed that of the 624 733 learners who appeared in matric examination in 2018, only 3856 learners had special educational needs, that is, 0.6% of the total (McKenzie et al. 2020). Another report by Watermeyer et al. (2013) reflected figures, which are higher in poorer provinces of South Africa; of those who do attend, most are still in separate, ‘special’ schools for disabled learners (https://static.pmg.org.za/170530report.pdf).

The main setbacks are experienced when implementing inclusive education at the community and school levels. It is a challenge whenever interventions are suggested for an education system that is void of inclusive approaches at community-school levels (Chataika et al. 2018). Funda (2020) believed that unless all teachers, teacher-administrators and non-disabled learners understand disability, disabled learners will be marginalised and stigmatised in mainstream schools because of a lack of political will from the government and support from the school authorities. Ndlovu (2020) shared a similar view although not exactly like Funda (2020) because Ndlovu (2020) did not raise political will in the narrative. Consequentially, disabled cohorts are commonly perceived as a source of extra costs within mainstream learning systems (Ostendorf 2020). On the one hand, parents at times contribute to dropouts because they remain overprotective of their vulnerable children regardless of the need to support them towards accessing education as a right (Ostendorf 2020). On the other hand, such behaviour is traceable to the socialisation (cultural background) that fosters a need to educate the parents and guardians on disability and inclusion for greater inclusive education (Ostendorf 2020). The author took cognisance of the findings mentioned here as not new but expressed differently by Funda (2020), Ostendorf (2020), and Turusida (2020). They interventions that can nurture every child to exercise the right to education regardless of having an impairment.

There is a shortage of literature on inclusive education, dropout amongst disabled learners, and opportunities to empower disabled learners for collaborative partnerships and participation in disability-inclusive community development. Furthermore, there is a limited framework, implementation matrix, and plan to support inclusive education practices in socio-economic and health-turbulence times (Monyane 2020). In addition, little is known on ways to manage fears of infection, fear of the unknown, and ignorance on how to handle disabled learners prevails among educators during COVID-19 pandemic, leading to their reduced educational success (Monyane 2020). However, there are teachers and community members who rely more on intuitive know-how as means to establish inclusion, cohesion, and integration for sustainable, inclusive community development and resilient education. Nonetheless, the above-mentioned situation or observable reality remains unaddressed because of a dearth of consultative dialogues to continuously gather opinions of (1) the community members (formal and informal support structures and systems), (2) disability activists, and (3) experts-based and research-based opinions of educationists, academics, and the disability sector in South Africa.

This article attempts to explore the underlying obstacles that induce school dropouts for disabled learners amidst the COVID-19 pandemic and in the aftermath of the said crisis. Interestingly, these struggles and obstacles are similar for all families with disabled children in South Africa (Ostendorf 2020). Therefore, this study can contribute to interventions that can alleviate dropouts for disabled learners within the education environment as an outcome of an applied research method presented in the next section.

## Methodology

Following Charmaz and Thornberg (2020), this study used a qualitative research approach. Interpretive and transformative research approaches enabled reality to emerge from the participants’ lived experiences as advised by Creswell and Poth (2018). The target population consisted of South African individuals. Purposive-stratified sampling recruited participants from Limpopo and Gauteng provinces because of the differences in dominating socio-economic activities and urban-rural settings. Gauteng is the most urbanised province, whilst Limpopo is dominated by rural areas in South Africa.

The participants comprised (1) parents or guardians of disabled children because they have lived experiences as part of the immediate family structure and support their child’s participation in learning and education; (2) The school leadership (heads) because they are the education administrators at the school level; (3) Social workers because they play a role in the well-being of vulnerable learners and their families in difficult times; while ensuring their safety, professional relationships and acting as guides and advocates; (4) Provincial Association of Persons with disabilities (APDs), who work closely with disabled children in the two provinces; (5) Office responsible for disabled learners in the Department of Basic Education custodians, policymakers and authorities in primary and secondary education; and (6) Office responsible for disabled children in the Department of Social Development because they are custodians and responsible for policies and authority on issues such as funding and resource support.

The research used a stratified-purposive sampling strategy to recruit participants who fall into different strata/categories as mentioned here and know the participation of disabled children in primary and secondary education as advised by Creswell and Poth (2018). Snowballing was used to recruit parents and guardians of disabled children in Limpopo and Gauteng provinces through the administrators of support groups. As a disability activist, the researcher has a work-based relationship with the disability sector – especially at the grassroots community level. The rest were recruited based on their office- job responsibilities whilst stationed in different organisations dealing with disabled learners’ participation in education.

The data were collected using an in-depth key-informant interview guide and focus group discussion (FGD) as advised by Alshenqeeti (2014). The FGDs verified and validated the data from participants, while in-depth-key informant interviews enabled individuals to share perspectives. The participants were asked questions in an attempt to address the overall research question. Participants were asked to: (1) describe or profile themselves and their learners; (2) describe their involvement and participation in the education of their disabled learners from enrolment, movement to and from school, learning, costs incurred for the child’s learning processes and cost of accessing education that is associated with the acquired impairment(s); (3) describe challenges or obstacles (causes of dropouts included) faced; and (4) describe ways to alleviate and improve disabled children’s participation in education.

The tools were pilot tested by three external people, namely, the head of a research department of one of the gatekeepers within the disability sector of South Africa, to check on political correctness and language used in the tools. An educator based in Zimbabwe and a disabled person who lives in South Africa conducted a peer review to check on the ability of the question to gather intended data as advised by Creswell and Poth (2018).

Data were collected after obtaining consent from participants using email and telephone calls because the research needed their lived experiences. Out of the proposed 30 parents and guardians of disabled learners in each province, only 17 from Limpopo province and 20 from Gauteng province gave consent and participated in the study. In addition, the data collection reached the heads of only two primary schools and one secondary school in Gauteng province. Only one primary school head from Limpopo gave consent and participated in the research. Only three social workers from Limpopo province and two social workers from Gauteng province gave participated by providing their opinions. Only one representative of associations of disabled children in the two above-mentioned provinces participated respectively. Only one person from the Department of Basic Education and the Social Development participated, respectively, thereby making the sample size to be N (50).

The researcher emailed participants who had consented to participate in the research and scheduled an appointment for the interview conversations and FGDs. The data collection used Google Meet to accommodate different impairments- communication and accessibility needs. All communication and discussions with all participants were conducted in the English language, which was their second language, and was comfortable to converse without an interpreter. All interviewees and the interviewer experienced no communication barrier. The data collection occurred between June and November 2021. Interviews and FGDs were opened by informed consent, which was read to the participants. Consent to voluntary participation and recording on the virtual platform was obtained before the conversations.

The collected data were analysed using Creswell’s (2014) qualitative data analysis framework. The gathered data were transcribed into text verbatim (Creswell 2014). Data were cleaned by reading several times whilst uncovering insights into the participants’ thoughts using inductive and deductive coding (Graebner, Martin & Roundy 2012). The author then organised and prepared a thematic analysis using a coding framework developed from the literature reviewed (theoretical framework) in this study (Eisenhardt cited in Gehman et al. 2018). Miles and Huberman (1994) advised the researcher to use descriptive coding by reading the transcribed data whilst assigning labels emerging from the gathered data, which attempted to answer this study’s research questions. The author used interpretive coding to fragment and reorganise data whilst identifying themes contextualised to the research and decontextualising the researcher’s experiences as advised by Miles, Huberman, and Saldaña (2013). Following Charmaz (2014), the author then read the codes and underlying data to find how the codes (themes and constructs) were categorised based on either thematic or conceptual similarities.

Following Saldaña’s (2015) advice, analytical memos were developed as the researcher’s ongoing reflections during the coding process. Such reflection was on the pattern of themes and codes and their interrelations as bridging the distinctions between coding, analysis, and results. Thus, Saldaña (2015) regarded memos as intuition, hunches, and serendipitous occurrences related to disability-inclusive education. The coding process and analytical memorising enabled the emergence of patterns in the data and did not determine the codes (Saldaña 2015). A computer-aid analytic tool-Atlas.ti.8 enabled the data to be immersed for in-depth analysis. This article identifies and elaborates new concepts and ideas that can enable sustainability and resiliency in disabled learners as a result of an inclusive education environment, thereby contributing to reducing disabled learner dropouts. A detailed description of the roles of individuals and organisations is presented as an element of a community-centric education-implementation plan to reduce dropouts amongst disabled learners. The role of the researcher cannot be ignored throughout the narrative; hence, the discussion articulates it as follows.

### Role of the researcher

The researcher’s reflexivity was part of the study as a disability advocate. However, audio-recording alleviated researcher biases during data collection as advised by Charmaz (2014). Following Charmaz and Thornberg (2020) and Creswell and Poth (2018), each group’s consolidated feedback was circulated to ensure trustworthiness and rigour through ascertaining the inclusion of all the opinions raised by participants. Coding fostered the anonymity of the participants from each forum, and the opinions that constituted the discussion were for this study only (Creswell 2014). Transcription was verbatim. However, the researcher’s influence during analysis and interpretation cannot be eliminated but mitigated by using computer-aided data analysis (Atlas.ti.8) (Creswell & Poth 2018). The researcher asked the administrators to delete the questions and responses posted online as agreed between the participants and the data collector as advised by Biriyai and Victor (2014). The results are as follows.

### Rigour and reliability

The collected data foster rigour and reliability because the data collection process followed the grounded theory assertion furthered by Charmaz and Thornberg (2020). Thus, Charmaz and Thornberg (2020) and Charmaz and Belgrave (2012) believed that a range between 10 and 30 in-depth interview participants’ opinions fosters data saturation level. Moreover, saturation was reached by the fourth in-depth key-informant interview and the second FGD. The rest of the conversations verified and confirmed a trend pattern (Gentles et al. 2019). At the same time, audio recordings of the interviews upheld the trustworthiness of the data as advised by Alshenqeeti (2014). The outcome of the analysis is presented in the Results section.

### Ethical considerations

Ethical clearance was obtained from the North-West University through the EMELTEN-REC. In addition, the authors underwent ethical training to research health and vulnerable groups. Authorisation and consent were obtained from participants before data collection and audio-recording (reference number: NWU-00248-18-A2, 23 August 2018).

## Results

Table 1 has the profile of the participants to have the contextual socio-economic background because it influences their views on school dropouts of disabled children. Ninety- five percent of the parents and guardians are females and 5% are males. Overall, the study has 50 participants, and female participants dominated with 90% against 10% of male participants. Ninety-two percent (92%) of the total number of parents and guardians are young single-black mothers aged between 25 and 40 years. However, 89% are dependent on South Africa Social Service Agency (SASSA) - Grants. Parents share similar narratives to Parent number 03, who said:

‘… due to my mother’s passing last year, I am now staying alone. I have no option except to quit work to take care of Thabiso (Pseudonym), and the father is not present in his life because he claims that it is not his child because of the impairment…’ (a mother of one disabled child, aged 35, lives in Limpopo).[in-depth interview which was conducted on the 3rd of July 2021].

**TABLE 1:** Profile of the participants.

Category and Total (*N*)	Type of engagement	Sex		Age	Marital status	Race	Education/range experience	Income range	Type of impairments	Age of the child
		Female	Male							
Parents *N* (33)-provide lived experiences with children with impairment(s).	In-depth individual interviews.	31	2	25–40	Single	Black	Matric and not employed (left work to take care of the child and fail to get employment)	South Africa Social Security Agency (SASSA) Grants (child-disability granted included)	Psychological disorders (5); Cognitive and Learning impairments (3); Hearing impairments (6); Vision impairments (8); Head Injuries – Brain impairment (1); Spinal Cord impairments (8); Mobility and Physical Impairments (6).	8–12 years
Guardians *N* (4)-provide lived experiences with a child with impairment(s).	Furthermore, four from each province participated in FGD.	4	-	25–40	Three married, one single	Black	Diploma and Degree; Employed	−300k annual		
Heads *N* (4)-teacher and management/administrative representative perspectives on inclusive education/approaches	One participated in in-depth individual interviews.	3	1	40–55	Married	Black	Diploma, Degree and Masters; Employed for 10–30 years	+300k annual	-	-
	Two participated in FGD in their respective provinces									
Social workers *N* (5)-provide perspectives on the well-being of vulnerable children with impairments and their families.	Two participated in FGDs in their respective provinces.	5	-	35–40	Three married, two singles	Two white, Indian, Two black	Degree; Employed for 10–15 years	+300k annual	-	-
	Three participated in in-depth individual interviews									
APDs *N* (2)-provide perspectives of activists and advocates for children with impairment(s).	Each participated in FGD in their respective provinces.	-	2	40–45	Divorce and married	Black	Diploma, Degree; Employed for 10–20 years	+300k annual	-	-
DBE *N* (1)-policymakers on disability-inclusive education	Participated in FGD for Gauteng province	1	-	45–55	Married	Black	Degree; Employed for 15 years	+300k annual	-	-
DSD *N* (1)-policymakers on resources support for disability-inclusive education	Participated in FGD for Gauteng province	1	-	45–55	Married	Black	Degree; Employed for 19 years	+300k annual	-	-

APD, Association of Persons with disability; FGD, focus group discussion.

However, the assertions cannot be generalised because contrasting accounts amongst participants show the desire to be employed to have resources that enhance provision for disabled children. For example, Parent number 10 said, ‘due to my child’s needs, SASSA grants are not adequate, yet I am failing to get employment…’ (a mother of two disabled children, aged 39 lives in Gauteng)[in-depth interview conducted on 27th of June 2021]. The majority of the parents and guardians are single parents who are active in the disabled child’s life.

Table 1 shows that different children at school going stage have acquired various impairments at birth. The parents’ narrative reflected that the impairments were diagnosed when the children were between 2 and 5 years. The impairments are classified into the following: psychological disorders (14%), cognitive and learning impairments (8%), hearing impairments (16%), vision impairments (22%), head injuries – brain impairment (2.7%), spinal cord impairments (22%) and mobility and physical impairments (16%).

Table 1 shows 12 participants in the age range of 35–55 years are neither parents nor guardians. Of the 12 participants, 9 are married, 1 is divorced and 2 are single and 6 are black females, 3 black males, 1 South African Indian and 2 white females. Furthermore, of these 12 participants, 2 had diplomas, and 9 had degrees and a master’s degree. The participants’ lived experiences ranged from 10 to 30 years. The participants’ annual income is above 3000 rands. Only 12 participants who were not in the parents and guardians stratum do not have children with impairments. The following section presents underlying obstacles that have contributed to the increase in dropouts of disabled learners.

### Challenges or obstacles

Literature shows anecdotal statistics of dropouts amidst the COVID-19 pandemic (Fodo 2020; Monyane 2020; UNICEF 2020). The given assertion concurs with accounts of five social workers, two APDs, and the representative office that handles disability issues for children in the Department of Social Development and Basic Education who participated in the study. Participants mentioned here believe that an inconsistent record exists that does not show disaggregated data on disabled children in terms of age, race, type of disability experienced, and type of impairment. The participants’ opinions on inclusion in education include misconceptions, misunderstanding, and misinterpretation of disability. The account of all participants but parents and guardians concurred that COVID-19 worsened the school attendance ratio amongst disabled children to the estimated percentage that is less than 10 as of the year 2021.

All parents and guardians had a similar view of being afraid of losing their learners to the disease. On the one hand, parents and guardians disagreed with the two government officials that ‘… when schools were opened, learners were aided to learn using virtual platforms’ [in-depth interview conducted on the 3rd of October 2021]. On the other hand, the parents’ and guardians’ narrative reflected their inability to mobilise resources to support learning at home. The majority of parents and guardians’ focus is on looking for means to afford food and other assistive devices to enhance the learners’ dignified living.

The results sum up key highlights that reflected challenges or obstacles that make the cohort more vulnerable to dropping out amidst the COVID-19 pandemic as follows:

1. Parents withdraw their children from early child development and primary education because mainstream schools do not have resource support for disabled learners, whilst school teachers lack the capacity and understanding to handle disabled children. For example, a case study of Parent number 05, an in-depth interview was conducted on 5th of August 2021:
  ‘Teachers and non-disabled learners mock (using words like suffering from … afflicted … unfortunate …). These words perpetuate disabled learners to feel alienated and not normal, thereby making learners feel uncomfortable to learn with disabled learners.’ (Parent of a disabled child, aged 37, who lives in Limpopo)
  Thereby, parents change schools and eventually pull out the child from school.
2. Parents are in a compromised situation that makes them enroll their children in mushrooming privately-owned special schools whose registration status is questionable. It is common that mainstream schools to reject their kids. Staff at schools ill-treated disabled pupils. For example, a case study of Parent number 01 from Focus Group Discussion 01:
  ‘When I went and asked for my child to spend a weekend at home from the boarding/school residential facilities, I noticed wounds that the principal failed to explain as such seem to have been caused by unattended diapers. Such schools have unqualified staff, dodge inspection from authorities until such cases are reported.’(Focus Group Discussion was conducted on 15 August 2021)
  Henceforth, fear, lack of trust, and parents’ insecurities contribute to dropouts. According to the parents and guardians’ account, rural areas have fewer mainstream schools that can reasonably accommodate disabled learners. A contrast emerges as private schools with all supportive online and physical learning systems are found in urban areas. The narrative of parents and guardians shows that they cannot afford the fees for their children to be enrolled in the urban special schools if one is not working for a salary above R150 000 per annum.
3. Heads/principals do not want to enroll the disabled learners at mainstream schools amidst this COVID-19 pandemic because they do not have adequate resources to include disabled learners.
  Head 001 said:
  ‘By having two separate policies for disabled and non-disabled learners, when staffing, we rarely think of considering the disabled learner in most cases. It rings a bell when we see a parent come with their child. That keeps us failing to build a system that systematically incorporates disability from the curriculum to practice.’ (Head of Primary School in Limpopo interviewed on 01 August 2021).
  Similar responses were given by two school heads from Gauteng, who added that primary and secondary schools do not have a Disability Unit to advise the administration on disability inclusion.
  All heads of schools in both provinces concurred that not all special schools have grades that uphold curriculum, and Care Centres are not suitable for every disabled learner without an official assessment. Thereby, making parents and guardians pull out their children as they are alienated and marginalised.
4. Two government officials proudly highlighted the milestones in facilitating the development of separate policies for disabled learners and non-disabled learners in mainstream schools. A contrasting view from parents and guardians reflected such milestones as perpetuating, if not causing dilemmas in establishing and communicating the roles of stakeholders in inclusive educated because educators neither understand disability nor have the capacity to conceptualise inclusion within the education environmental setting interprets disability policy at the school level.
5. Social workers and heads of schools concurred that there is a misconception, misunderstanding, and misinterpretation of disability and inclusion among education researchers. The participants who were neither parents nor guardians in FGD 01 concurred that dropouts during the COVID-19 pandemic were worsened by the lack of clarity on the disability policies, poor policy implementation of the said policies at the district, community, and school level, and lack of measurement and enforcement on inclusive education.

### Implications and interventions

The suggestions that were gathered from participants highlighted the need to have a clear stakeholder-role definition in inclusive education and their participation in the reduction of dropouts of disabled learners. This is illustrated in Table 2.

**TABLE 2:** Roles of stakeholders in inclusive education for the educational success of disabled children.

Stakeholders	Roles	Activities	Method
Disabled learner	Aware of differences amongst each other and such is part of normalcy	Interactions within shared learning space and playing area.	Play and learn together in one shared space. Make learners self-reflect and share how they feel about disengaging with the support structure.
Teachers/educators	Teach learners to be aware of differences and those differences do not have to make them uncomfortable. Attend all training sessions or workshops relating to absenteeism tracking and dropout. Implement all processes relating to absenteeism tracking in the classroom. Recognise the value of taking daily attendance. Keep records up to date. Follow up, with absent learners.	Avoid words that mock impairment or disability, e.g. afflicted, suffering or unfortunate… Share observed signs of disengaging and distress among learners with responsible authorities.	Collect and submit class-level data on absenteeism and disengagement (e.g. attendance registers). Understand teachers’ value and critical role in gathering and recording accurate attendance data. Monitor and use the given tools to track red flags. Timeously report red-flagged learners – absent, disengaged, or showing behavioural issues.
Teaching management and school governing board/Community-based school development council (at the institution)	Create inclusive teaching and learning (including content, delivery, and outcomes).	Develop and implement dropout proactive and reactive interventions frameworks and tools guided by policy.	Develop a process to train teachers and hold them accountable. Develop tools and forms for all teachers to use in absenteeism tracking. Discuss and analyse absenteeism and dropout regularly.
Teaching management at the district level and school level	Develop a tracking system for schools in the district	Train schools in using tracking/early warning and data analysis tools. Feed school-level early warning data into a more comprehensive analysis at the district level to identify broader patterns.	Encourage the schools in the district to adopt absenteeism tracking processes. Develop and supply tools to assist schools in collecting absenteeism data efficiently. Support schools to be able to gather absenteeism data effectively
Government authorities and agents (at the national, provincial, and district levels)	Establish enforceable legislative tools with adequate resources and policies and implementation frameworks. Establish consultative and implementation committees composed of multi-stakeholders. Establish an inter-ministerial monitoring and evaluation council that liaises with all stakeholders.	Establish enforceable legislative tools with adequate resources and policies and implementation frameworks. Establish consultative and implementation committees composed of multi-stakeholders. Establish an inter-ministerial monitoring and evaluation council that liaises with all stakeholders.	Establish enforceable legislative tools with adequate resources and policies and implementation frameworks. Establish consultative and implementation committees composed of multi-stakeholders Establish an inter-ministerial monitoring and evaluation council that liaises with all stakeholders.
Support Structure at household (family, parents, siblings, and caregivers/guardians), learner representatives, community, Disabled people organisations (DPOs) for children, Children Rights Organisations/ Civic organisations advocating for children’s rights.	Help collect, collate and manage data on absenteeism and disengagement. Identify and verify various context-specific early warning signs that can be monitored using data.	Develop tools or methods or offer resources that directly assist with absenteeism tracking. Work with key bodies in the school such as learners’ representatives, school development council/board, parents etc., and train them on the value and best approaches to tracking absenteeism.	Provide technical support for managing and analysing attendance and dropout risk data. Provide the technology/technical support needed to gather and analyse data or make the prevention work. Support for joint-collaborative partnerships with schools towards the implications of the analysed data on approaches and policies.

**FIGURE 1:**
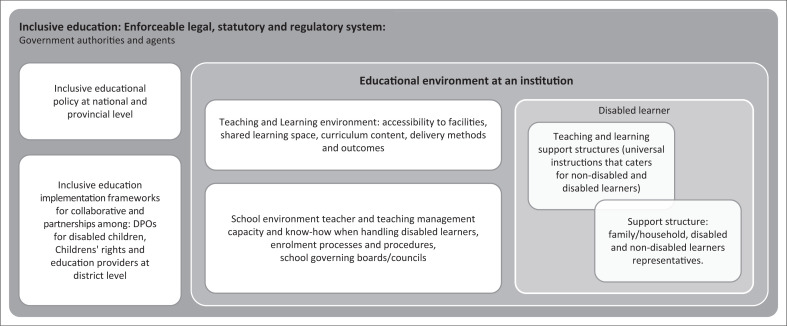
Role-players for the educational success of disabled learners.

Based on the participants’ suggestions and deductions from the pattern of views, Table 2 and Figure 1 present the recommendations and the usage of a systematic multistakeholder local community-based intervention approach. This would enable continuous interactive relationships and meaningful engagement and dialogues amongst role-players to reduce dropouts whilst enabling the educational success of disabled learners.

A total of 50 participants suggested that if the role mentioned here can be available and active, dropouts amongst disabled learners can be reduced and prevented (see Table 2; Figure 1).

### Implementation of the roles of the role players

Based on Figure 1, a national and provincial action plan should inform the development of a toolkit to be implemented at a local community-school level. A joint community-based action plan can embed the participation of every member of the community’s population groups in (1) problem identification, (2) co-designing of methods of investigating contextual obstacles and toward greater understanding of disability and participation of disabled children in education, and (3) co-designing roles and assigning roles in implementing interventions on dropouts, and education and raising awareness to all community members on ways to mitigate dropouts amongst disabled learners.

Based on Table 2 and Figure 1, the following 10 focal activities should be considered when implementing the intervention: (1) identifying; (2) acknowledging obstructs faced by disabled learners; (3) understanding disability and know-how when handling disabled learners; (4) inclusive education environment (policies, implementation frameworks that foster resource allocation and adequate capacity); (5) inclusive enrolment processes, procedures and learners’ retention systems within teaching and learning; (6) school attendance (virtual and physical) and observation of disabled learners’ learning behaviours and signs of disengaging; (7) parents and teacher collaborative partnership in facilitating reasonable accommodation in the classroom as also suggested by Mudzingwa (2020) that family is the critical support system for resilience that aids to personal and internal resilience of a disabled person, by Daly (2020) that inclusion is influenced by closeness to vital resources so that it builds resilient and well-being, as found by Le Roux (2018) that internal resilience makes disabled individuals strive to learn new social and surviving skills to sustain themselves against the adverse odds; (8) psychosocial support systems such as identifying and strengthening community network support-group just as proposed by Mudzingwa (2020); (9) collaborative partnership development with clear role definitions and communication system; and (10) tracking and monitoring disabled learners who dropped out and those who completed school so that there is a continuous appropriate engagement during the roll-out of interventions. One should take note that often, disabled learners are told by societal structures, who they are, and what to believe about themselves. The conclusions derived from the findings are discussed in the next section.

## Conclusion

**FIGURE 2:**
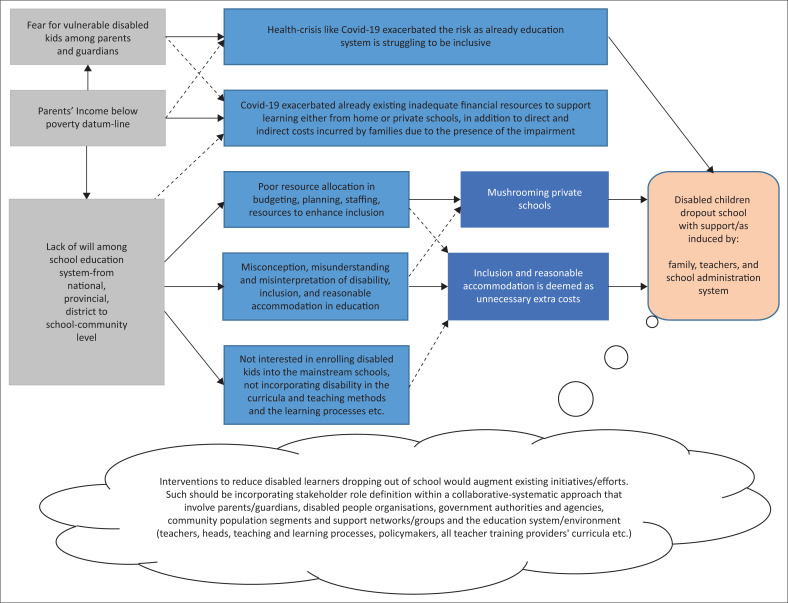
Summary of underlying obstacles that lead to dropping out of school and postulated interventions.

Based on the given discussion, it is clear that a systematic multistakeholder local community-based intervention approach can contribute to an effort that can improve the educational success of disabled learners. Therefore, the author concludes that dropouts amongst disabled learners can be reduced if obstacles are acknowledged and then contextualise the interventions within the socio-economic setting of communities where the schools are located, as summarised in Figure 2.

The factors in brown colour are the leading causes that lead to obstacles in blue colours. The broken arrows show the indirect connection between the factors; however, the unbroken arrow directly influences identified moderating and intermediating factors. The factors in orange are the outcome of the interaction of the factors highlighted in this study. The cloud represents the postulated interventions that augment existing initiatives to reduce dropouts amongst disabled learners. Hence, it is essential for the two responsible government authorities to plan, budget, bring more human capital resources and develop a national toolkit that guides stakeholders’ roles at provincial, district, and local community-based school levels. It is ideal when a disability-inclusive national action plan explicitly aims to prevent dropouts amongst disabled learners. Therefore, the study attempts to contribute with an intervention that can facilitate reducing systemic ignorance, misconception, misunderstanding, and misinterpretation of disability and inclusion to the extent that guardians and parents initiate the dropouts. Nonetheless, the study was limited to a qualitative approach that did not cover all provinces. Hence, future studies are recommended to have a longitudinal approach whilst having a wider cross-sectional coverage within the borders of South Africa.
